# Effect of Partnership Status on Preferences for Facial Self-Resemblance

**DOI:** 10.3389/fpsyg.2016.00869

**Published:** 2016-06-14

**Authors:** Jitka Lindová, Anthony C. Little, Jan Havlíček, S. Craig Roberts, Anna Rubešová, Jaroslav Flegr

**Affiliations:** ^1^Department of Anthropology, Faculty of Humanities, Charles UniversityPrague, Czech Republic; ^2^National Institute of Mental Health, Charles UniversityPrague, Czech Republic; ^3^School of Natural Sciences, University of StirlingStirling, UK; ^4^Department of Zoology, Faculty of Science, Charles UniversityPrague, Czech Republic; ^5^Department of Philosophy and History of Science, Faculty of Science, Charles UniversityPrague, Czech Republic

**Keywords:** self-resemblance, facial attractiveness, relationship status, mate choice, disassortative mating

## Abstract

Self-resemblance has been found to have a context-dependent effect when expressing preferences for faces. Whereas dissimilarity preference during mate choice in animals is often explained as an evolutionary adaptation to increase heterozygosity of offspring, self-resemblance can be also favored in humans, reflecting, e.g., preference for kinship cues. We performed two studies, using transformations of facial photographs to manipulate levels of resemblance with the rater, to examine the influence of self-resemblance in single vs. coupled individuals. Raters assessed facial attractiveness of other-sex and same-sex photographs according to both short-term and long-term relationship contexts. We found a preference for dissimilarity of other-sex and same-sex faces in single individuals, but no effect of self-resemblance in coupled raters. No effect of sex of participant or short-term vs. long-term attractiveness rating was observed. The results support the evolutionary interpretation that dissimilarity of other-sex faces is preferred by uncoupled individuals as an adaptive mechanism to avoid inbreeding. In contrast, lower dissimilarity preference of other-sex faces in coupled individuals may reflect suppressed attention to attractiveness cues in potential alternative partners as a relationship maintenance mechanism, and its substitution by attention to cues of kinship and psychological similarity connected with greater likelihood of prosocial behavior acquisition from such persons.

## Introduction

The choice of a mating partner depends not only on characteristics of the potential partner, but also on the degree to which these characteristics complement the choosing individual. Although this principle applies to varied traits, including personality and socio-cultural characteristics, it is especially apparent in inbreeding avoidance, where individuals avoid mating with close relatives. In humans, this is known as the “Westermarck effect” (Wolf, 1995; Rantala and Marcinkowska, 2011). In biological terms, incest avoidance is beneficial because it reduces the likelihood that offspring are homozygous for recessive genes carrying deleterious or even lethal mutations (Brown, 1997). In addition to cultural incest taboos, a further mechanism to efficiently prevent mating with unknown kin is disassortative (or negative assortative) mating, for example through low attraction toward genetically similar individuals (e.g., Roberts and Little, 2008; Havlicek and Roberts, 2009). This can bring additional benefits at the biological level, because disassortative mating increases heterozygosity in resulting offspring, which can lead to increased viability (e.g., higher disease resistance; Brown, 1997). In animals, disassortative mating preferences are commonly described across taxa and usually mediated by odor cues (Penn, 2002). Some evidence suggests that humans also express disassortative odor preferences, at least under certain conditions (Havlicek and Roberts, 2009).

Despite these potential biological benefits, studies on human mate choice demonstrate that, with the possible exception of odor preferences, positive rather than negative assortative preferences are the rule: many studies show morphological (Zajonc et al., 1987; Bereczkei et al., 2002; Little et al., 2006) as well as psychosocial (Keller et al., 1996; Buston and Emlen, 2003) similarity within couples (also termed ‘homogamy’). This is no less the case in terms of facial preference. Research shows that individuals prefer faces of unfamiliar individuals who are genetically similar (Roberts et al., 2005), and experiments using morphing techniques to manipulate facial shape also typically report preference for similarity, not dissimilarity, in opposite-sex faces (Penton-Voak et al., 1999; DeBruine, 2004; Saxton et al., 2009; DeBruine et al., 2011; Kocsor et al., 2011; Watkins et al., 2011). Similarity preferences extend to actual choice of facially similar individuals (Griffiths and Kunz, 1973; Hinsz, 1989). Positive assortative mating has been explained in terms of several psychosocial benefits, such as higher satisfaction in long-term couples with similar traits, including personality, attitudes, values, and intelligence (Klohnen and Mendelsohn, 1998; Watson et al., 2004; Luo and Klohnen, 2005; Štěrbová and Valentova, 2012). It could also help to prevent disruption of coadapted gene complexes, i.e., sets of genes whose effects are facilitated by interactions among them (Read and Harvey, 1988). Preference for facial self-resemblance further likely derives from associations between self-resemblance and perceived levels of future social support, because individuals receive greater support from kin compared to non-related individuals (DeBruine et al., 2005). These associations are likely to develop early in life, through sexual imprinting-like processes whereby an individual comes to prefer potential mates resembling his/her other-sex parent (Bereczkei et al., 2004). An alternative mechanism is self-referent phenotype matching, in which individuals prefer those who resemble themselves, rather than kin (e.g., Bressan and Zucchi, 2009).

Using experimental manipulation of facial images, some authors intended to differentiate the opposite tendencies toward disassortative and assortative mating by contrasting the self-resemblance effect on short-term vs. long-term facial attractiveness ratings. Physical attraction is considered to be the dominant criterion for mate choice in the short-term context (Gangestad and Simpson, 2000) potentially leading to preference for cues of genetic dissimilarity, and psychological benefits associated with assortative mating were considered to play a greater role when looking for a long-term partner leading to preference for similarity cues (Trivers, 1971). DeBruine (2005) indeed showed that in the short-term but not in the long-term mating context, self-resemblance moderately decreased attractiveness ratings of opposite-sex faces. However, Saxton et al. (2009) conversely found a preference for self-resembling faces in the short-term, but not the long-term context. Overall, the effect of the (instruction based) short-term vs. long-term attractiveness distinction when assessing self-resembling/dissimilar faces is ambiguous. It might be that this approach suffers from low external validity when it requires that participants concentrate on different aspects of visual attractiveness of a face solely on the basis of different verbal instructions. In consequence, participants may rate facial attractiveness identically in both cases, or tend to take other than physical (e.g., social) cues into account when performing the long-term attractiveness ratings, as was shown by Little et al. (2008; see also: Confer et al., 2010).

It seems more efficient to employ participants’ biosocial contexts to investigate the contrasting effects of disassortative and assortative mating preferences. For example, DeBruine et al. (2011) showed that having male brothers increased women’s aversion to self-resembling opposite-sex faces, possibly due to stronger learning or motivation for incest-avoidance in women with many opposite-sex kin in her childhood surroundings. A positive effect of self-resemblance on facial preferences was found in women with a positive emotional relationship with their father (Watkins et al., 2011), and preference for facial similarity was stronger during high progesterone phases of the menstrual cycle (DeBruine et al., 2005).

In addition, previous studies have indicated that a positive effect of self-resemblance on facial preferences exists for same-sex but not other-sex faces (DeBruine, 2004), and is stronger when women rate relatively masculinized compared to femininized male faces (Saxton et al., 2009).

### Current Study

One factor that, to date, has not been explored with regard to self-resemblance effects on facial preferences is the (long-term) partnership status of participants. On the basis of existing theory and evidence, we can formulate two opposing predictions regarding the effect of partnership status on self-resemblance preferences.

According to the one line of evidence, coupled and non-coupled individuals differ in their attentiveness to cues of sexual attractiveness; whereas non-coupled individuals are likely to consider sexual attraction strongly when judging others because they are more actively mate-searching, coupled individuals are less likely to be attentive to cues of sexual attractiveness. Romantically involved subjects were found to spend less time observing attractive opposite-sex individuals (Miller, 1997), pay less visual attention to alternative partners (Maner et al., 2008; Koranyi and Rothermund, 2012), show a lower differentiation between attractive and non-attractive individuals in the visual (Karremans et al., 2011) and olfactory domains (Lundström and Jones-Gotman, 2009), and rate attractiveness of opposite-sex strangers lower than controls (Simpson et al., 1986). Koranyi and Rothermund (2012) conclude that reciprocal romantic interest eliminates the automatic attentional bias toward attractive opposite-sex faces. In addition, it was suggested that coupled individuals are more likely to derogate the attractiveness of others as an adaptive relationship maintenance mechanism (Maner et al., 2009). Following this line of argument, we propose that being in a romantic relationship also reduces the perceptual preference for dissimilar faces, which is in single individuals proposed to serve as a mechanism to avoid mating with kin and increase levels of heterozygosity of potential offspring. In contrast, among coupled participants, other cues provided by facial appearance not directly related to mate-choice might become more important. First, cues of kinship are likely to be preferred because of their link with expectations of prosocial behavior (Trivers, 1971). Field studies often show that support from kin is a dominant factor at least for women during their long-term partnership and regarding their reproductive success (e.g., Bereczkei, 1998; Leonetti et al., 2004). And second, cues of self-resemblance can be also favored in others reflecting the tendency to positive assortative pairing when choosing friends (Cohen, 1977; Kandel, 1978). In summary, this line of reasoning would lead to the prediction of a preference for dissimilarity when judging facial attractiveness in single participants, but to a preference of self-resemblance in coupled participants. (Note that this prediction is independent of the short- vs. long-term mating context distinction, and is expected to have a stronger effect than the instruction-based distinction of short- vs. long-term context, as it is based on proposed actual perceptual changes in coupled vs. single participants instead of struggling to provoke such changes through verbal instruction).

According to the other line of reasoning, however, being partnered vs. single and looking for a short- vs. long-term partner is strongly linked and leads to a contrasting prediction of higher dissimilarity preference in coupled than in uncoupled. Coupled individuals might be especially attentive to cues of genetic quality and compatibility because they may seek attractive extra-pair partners (who are argued to have “good genes”) who are not expected to provide any paternal care (see e.g., Gangestad and Thornhill, 2008). Thus, the interest in facial cues of genetic quality may be relatively high, compared to singles (who are, somewhat speculatively, proposed to look for a long-term partner in the first place). Evidence consistent with this interpretation includes a greater preference for male masculinity among coupled regularly cycling women compared with uncoupled women (Little et al., 2002).

The first aim of our study was to test between these two lines of reasoning, where the former one leads to a prediction of facial similarity preference in coupled individuals and facial dissimilarity preference in singles, and the latter one leads to an opposite prediction of higher dissimilarity preference in coupled than uncoupled participants. In addition, we predicted higher preferences for dissimilar faces within the short-term relationship context than within the long-term relationship context, because the long-term context is associated with additional advantage of assortative mating with a partner with similar psychological characteristics (Luo and Klohnen, 2005). We expect, however, this difference to be relatively weak, if any, as previous research looking at the effect of self-resemblance on short- vs. long-term attractiveness judgments brought inconsistent results, and because of the concerns regarding low external validity of distinguishing between the short- and long-term contexts on the basis of verbal instruction only.

## Study 1

### Methods

#### Participants

Participants were 91 female and 29 male (age 21–31 years, mean 24.5, *SD* = 2.0) students of the Faculty of Science at Charles University, Prague. They were contacted by email, and offered a 200CZK (approximately 10 USD) reimbursement for taking part in the study. A facial photograph was obtained as a part of a separate study (Lindová et al., 2010) so that participants did not associate the current study with the previous photograph acquisition. During photographing, participants were instructed to look into the camera and maintain a neutral facial expression. All participants provided an informed consent with the use their photograph for scientific purposes. Attractiveness ratings of these photographs were obtained for another study, as a mean of *z*-scores of ratings of his/her photograph by five female and six male students from another faculty. The sample size that we reached is comparable to sample sizes used in similar studies on facial perception which found significant results (e.g., DeBruine, 2004, 2005; DeBruine et al., 2005; see also **Table 1** for numbers in all categories).

**Table 1:** Numbers of participants in categories compared by generalized linear models (GLM).

Category of participants	
**Study 1**	
Coupled men	18
Single men	11
Coupled women	64
Single women	25
**Study 2**	
Coupled men	24
Single men	10
Coupled women	73
Single women	30
**Studies 1 and 2 combined**	
Coupled men	17
Single men	7
Coupled women	51
Single women	14


#### Stimuli

**FIGURE 1:**
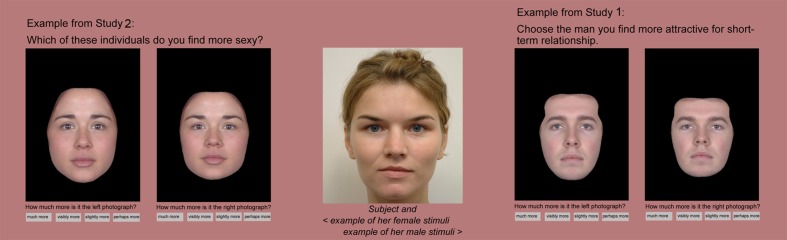
**Examples of stimuli.** In the center is a neutral face image of one of the participants. On the left is an example of a same-sex pair of face transforms. The base face to which the transforms are applied is a composite of three other faces. The self-resembling transform from each pair was made by applying 50% of the shape difference between the participant’s face and the same-sex composite face to a target’s photograph. To make the non-self-resembling transform, a reversal of this 50% difference was applied. On the right is a similarly constructed opposite-sex pair of transforms. Within each pair of transforms, the transform resembling the rater is on the right.

First, we created a set of unfamiliar base faces, using neutral-expression photographs of 45 female and 45 male Caucasian students from the University of Liverpool. All participants provided an informed consent with the use their photograph for scientific purposes. Women were asked not to wear make-up and all men were asked to be shaved on the day of taking photographs. Subsequently their hair, ears, neck, and all visible parts of clothing and background were digitally masked, leaving only internal facial features available for rating. To reduce the effect of extreme facial features on rating, we then created 15 female and 15 male composites, each composite being made from three same-sex photographs. This was done with the image manipulation software Psychomorph (Tiddeman et al., 2001) after delineating the shape of the face using 171 facial landmarks and combining the shape and texture of the pictures. Because we used only a small number of photographs for each composite, this process retained a highly realistic appearance in the target images (see **Figure 1**).

The shape of each of the Czech participants’ faces was also delineated in Psychomorph, using the same 171 facial landmarks as for the base faces. Subsequently, each participant’s photograph was used to make shape-only transforms of the target composite faces. Shape-only transforms preserve the color information from the original target image, but focus on shape as a cue to genetic similarity. Shape-only transforms were used in several previous studies on self-resemblance and mate preferences (Penton-Voak et al., 1999; DeBruine, 2004, 2005; DeBruine et al., 2005). Transforms were made by calculating the shape differences between each participant’s face and the same-sex composite faces. The “similar-transform” was made by applying 50% of this difference to the target composites. The “dissimilar-transform” was made by applying the reverse of this 50% difference to the composites. The resemblance/dissimilarity manipulation of 50% was chosen because it was considered to be too subtle to be noticed by the raters (DeBruine, 2005). For each participant, 30 pairs of transforms were obtained, where each pair consisted of one “self-similar transform” and one “self-dissimilar transform” of the same target composite, and where 15 pairs were of the same-sex, and 15 of the opposite-sex, to the participant. Participants were unaware that the transforms had been made using their own face shape.

#### Procedure

Each participant was provided with a personal login and password for the internet application where ratings were to be conducted, where they had access to a questionnaire and the specific set of photographs created for them (his/her self-resembling and dissimilar morphs). After logging in, participants were first asked to indicate whether or not they currently had a romantic partner.

The sets of 15 same-sex and 15 opposite-sex pairs of self-similar and self-dissimilar transforms were presented to each rater. Raters were asked to choose which of the two image versions they preferred (e.g., according to attractiveness) and also to indicate the strength of this preference from one of four options (*perhaps more, slightly more, visibly more, much more*; **Figure 1**).

Verbal descriptions of short-term and long-term romantic relationships were used to provide a standardized context for attractiveness ratings. These were adapted from the study of Little et al. (2007). The two instructions for attractiveness ratings of the opposite-sex were as follows: “Choose the man/woman (according to the sex of rater) which you find more attractive for a short-term/long-term romantic relationship.” The instructions for same-sex attractiveness ratings also had two variants: “Choose the man/woman (according to the sex of rater) which you think will be more attractive to the opposite sex” and: “Choose the man/woman (according to sex of rater) which you would prefer as your friend.” To distract attention between subsequent ratings of attractiveness of the same targets in different contexts, we also asked raters to rate the trustworthiness of the targets (“Choose the man/woman which you find more trustworthy”). The final sequence of ratings was as follows: (1) opposite-sex targets, short-term attractiveness, (2) same-sex targets, trustworthiness, (3) opposite-sex targets, long-term attractiveness, (4) same-sex targets, attractiveness to the opposite sex, (5) opposite-sex targets, trustworthiness, (6) same-sex targets, preference as friends.

The experimental procedure conforms to the legislation of the Czech Republic and was approved by Institutional Review Board Charles University, Faculty of Sciences, and by the University of Liverpool Committee on Research Ethics.

#### Statistical Analyzes

We calculated preference for self-resemblance by combining the two scales from the pair of transforms to form one 8-point scale, where the choice *much more* for the self-dissimilar-transform was assigned to point 1, the choice *perhaps more* for the self-dissimilar-transform was assigned to point 4, the choice *perhaps more* for the self-similar-transform was assigned to point 5, and the choice *much more* for the self-similar-transform was assigned to point 8 (remaining choices were assigned respective intermediate points). According to this procedure, an average score of 4.5 across all pairs would correspond to random choice between pairs of transforms.

To test the effect of sex and partnership status on self-resemblance preference, we performed a set of generalized linear models (GLM) in four attractiveness rating contexts (short-term and long-term attractiveness for same-sex and opposite-sex faces) and two control rating contexts (trustworthiness for same- and opposite sex faces). In all models, we used as categorical predictors the sex of rater and their partnership status (for numbers of participants in all categories, see **Table 1**) and the attractiveness of rater as a covariate. We controlled for the effect of rater attractiveness because coupled women raters were scored as having higher attractiveness than single women raters (*t*_87_ = -2.76, *p* = 0.007, 95% CI [-0.68, -0.11]; coupled and single men did not differ in attractiveness: *t*_27_ = 0.13, *p* = 0.90, CI [-0.40, 0.45]).

Ratings (averaged for each rater) of each characteristic (attractiveness in long-term context, trustworthiness etc.) were additionally compared using one-sample *t*-tests against the criterion of 4.5 (random choice) to test the potential effect of self-resemblance on each particular rating.

All statistics were performed in SPSS 16.

### Results

Across the whole sample of raters, we found no overall preference for either self-resembling or self-dissimilar faces when rating same-sex and opposite-sex photographs in either one of the rating contexts (short-term/long-term attractiveness, trustworthiness; average ratings 4.45 to 4.49, *t*_119_ = -0.13 to -0.64, all *p* > 0.61). Attractiveness of rater had a positive effect on self-resemblance preference in one rating context, namely when rating same-sex images as they would be seen by the opposite sex (*F*_1,113_ = 7.87, *p* = 0.007, η^2^ = 0.064).

#### Partnership Status

The effect of partnership status on self-resemblance preference was relatively stronger in the case of the ratings of short-term attractiveness of opposite-sex photographs (*F*_1,113_ = 3.18, *p* = 0.077, η^2^ = 0.027). In contrast, partnership status and sex of participant had no significant effect on other ratings (all *F*_1,113_ < 2.5, all *ps* > 0.12, η^2^ < 0.015).

*Post hoc t*-tests revealed that within the short-term attractiveness context, uncoupled raters tended to prefer dissimilar opposite-sex faces (mean rating = 4.20, *t*_35_ = -1.98, *p* = 0.056, CI [-0.60, 0.01]), whereas coupled raters did not show either a preference for self-resemblance or dissimilarity in this context (4.57, *t*_83_ = 0.67, *p* = 0.50, CI [-0.14, 0.27]).

#### Short-Term vs. Long-Term Mating Context

To investigate this further, a repeated measures GLM was performed to test for a possible effect of the mating context (short-term vs. long-term) on self-resemblance preference in attractiveness ratings of opposite-sex photographs. As in previous GLM analyses, we controlled for sex, partnership status and attractiveness of rater. However, we found no effect of the mating context (*F*_1,116_ = 1.27, *p* = 0.26, η^2^ = 0.011).

## Study 2

In Study 1, we had participants rate preferences according to short-term vs. long-term relationship contexts, which is a method widely used in the human mate preferences literature (e.g., Little et al., 2007). These two contexts tend to produce differences in expressed preferences. Whereas for short-term relationships physical attractiveness is the most valued mate characteristic by both men and women, and physical attraction the most likely reason to enter such a relationship, other characteristics play a relatively more important role in long-term relationships. Although men tend to consider physical attractiveness as relatively important in the long-term context, women value status and resources relatively more. In addition, both sexes rank kindness/warmth and intelligence among the two most important characteristics in long-term mates (Regan and Berscheid, 1997; Regan, 1998; Buunk et al., 2002; Fletcher et al., 2004; Li and Kenrick, 2006). It is not known, however, whether people cross-culturally spontaneously make such a distinction or if, having been externally imposed by researchers, it might therefore be cognitively demanding for the participants, at least in cultures without a strong sexual hookup tradition (Garcia et al., 2012). In Study 2, we intended to circumvent the uncertainty connected with the use of verbal depictions of short- and long-term mating contexts by using instead specific adjectives that represent key preferences in these contexts. Thus we use the adjective “sexy” to represent the physical attractiveness preference within the short-term context, and “nice” 1 representing the kindness/warmth preference within the long-term context, and we also compare their use with the more classical verbal depiction used in Study 1. (In Czech, the adjectives “sexy” and “sympatický” were used.)

### Method

#### Participants

The same target faces as in Study 1 were used. Raters from Study 1 also served as raters in Study 2, with some additional raters recruited. The total number of raters was 106 women and 33 men.

#### Procedure and Stimuli

The experimental procedure was similar to Study 1, including rating instructions (“Choose the man/woman which you find more …”), scoring (choosing which of the two image versions they preferred and indicating strength of this preference), but raters judged according to the adjectives “sexy” and “nice” instead of the short-term and long-term mating context. As in Study 1, trustworthiness was also rated. The sequence of rating for both female and male raters was as follows: (1) female targets, sexy, (2) male targets, sexy, (3) female targets, nice, (4) male targets, nice, (5) female targets, trustworthy, (6) male targets, trustworthy.

#### Statistical Analyzes

Scores for rated preferences were combined as in Study 1 (forming an 8-point scale of self-resemblance preference with the average of 4.5). As in Study 1, GLMs were performed for each rated characteristic to test the effect of sex and partnership status on self-resemblance preference. Again, attractiveness of rater had an effect on self-resemblance preference in some rating contexts, namely when rating sexiness of same-sex faces (*F*_1,131_ = 9.42, *p* = 0.003, η^2^ = 0.067), “nice” in opposite-sex faces (*F*_1,131_ = 5.48, *p* = 0.021, η^2^ = 0.040), “nice” in same-sex faces (*F*_1,132_ = 7.23, *p* = 0.008, η^2^ = 0.052), and trustworthiness in opposite-sex faces (*F*_1,132_ = 6.47, *p* = 0.012, η^2^ = 0.047). Therefore, attractiveness was included in the model. As in Study 1, *t*-tests were performed to compare ratings of each characteristic (sexiness, trustworthiness etc.) against the criterion of 4.5 (random choice).

### Results

Across the whole sample, self-resemblance had no significant effect on the ratings of how “sexy,” “nice,” and “trustworthy” the opposite or same-sex faces appeared: all average ratings were between 4.44 and 4.54 (*t*_138_ = -0.73 to 0.67, *p* > 0.46).

#### Partnership Status

The effect of partnership status on self-resemblance preference in the context of sexiness ratings of opposite-sex photographs was not significant, although the effect was close to *p* = 0.05 (*F*_1,132_ = 3.27, *p* = 0.073, η^2^ = 0.024), but we found a significant effect of partnership status on self-resemblance preference when rating same-sex sexiness (*F*_1,131_ = 5.49, *p* = 0.021, η^2^ = 0.040). There was no effect of partnership status on self-resemblance preference in the context of ratings of how nice the opposite-sex (*F*_1,131_ = 2.36, *p* = 0.13, η^2^ = 0.018) or same-sex person appears (*F*_1,132_ = 0.73, *p* = 0.39, η^2^ = 0.006). Sex of participants had no effect on self-resemblance preference.

*Post hoc t*-tests showed that single raters judged dissimilar opposite-sex faces as more sexy (mean rating 4.24, *t*_39_ = -2.05, *p* = 0.047, CI [-0.51, 0.00]) than self-resembling opposite-sex faces. They also rated dissimilar same-sex photographs as more sexy (mean = 4.11, *t*_39_ = -2.63, *p* = 0.012, CI [-0.69, -0.09]) than self-resembling same-sex photographs. Scores for the sample of coupled participants did not differ from chance (4.58 and 4.58, *t*_97_ = 0.93 and 0.80, *ps* = 0.35 and 0.42, CI [-0.08, 0.26] and [-0.11, 0.26] for sexiness ratings of opposite- and same-sex faces, respectively).

#### “Sexy” vs. “Nice” Rating Context

A repeated measures GLM was performed to test for a possible effect of the rated characteristic (sexy vs. nice) on self-resemblance preference in opposite-sex photographs, controlling for sex, partnership status and attractiveness of rater. We found no effect of the repeated measure rated characteristic (*F*_1,131_ = 0.01, *p* = 0.91, η^2^ < 0.001). Instead, partnership status was a significant predictor of the model (*F*_1,131_ = 3.90, *p* = 0.050, η^2^ = 0.029).

#### Combined Analysis of Studies 1 and 2

Finally, we combined corresponding ratings from each rater in both studies, excluding 28 raters who changed partnership status between the two ratings. The main aim of this analysis was to test if our new methodology used in Study 2, namely rating how “sexy” and “nice” a person appeared, produces different results when compared with the often used attractiveness rating in verbally described hypothetic short-term or long-term contexts from Study 1.

**FIGURE 2:**
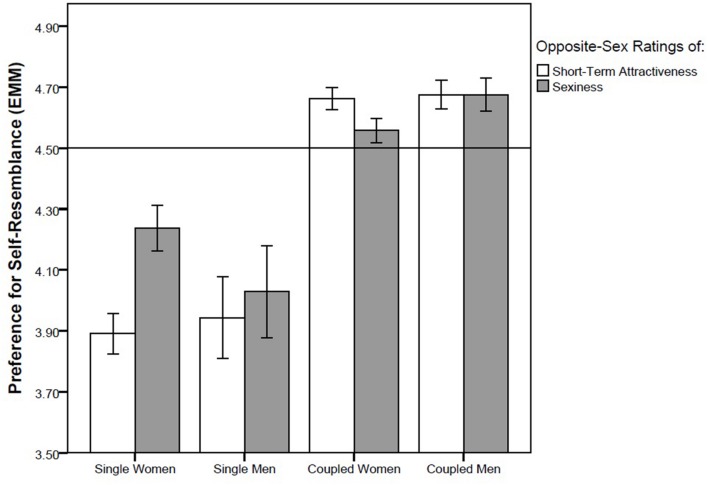
**Self-resemblance preference in ratings of short-term attractiveness and sexiness in opposite-sex faces.** Preference score higher than 4.5 (depicted by a horisontal line) means that the self transform was on average preferred over the non-self transform. Error bars represent 95% CI. EMM … Estimated Marginal Means.

**FIGURE 3:**
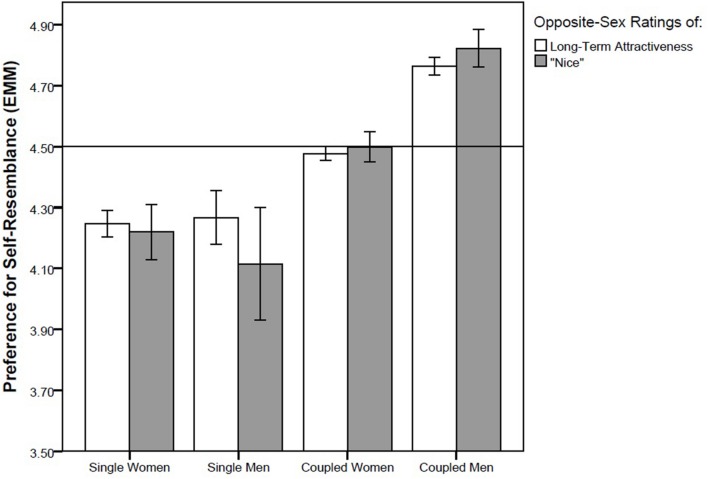
**Self-resemblance preference in ratings of long-term attractiveness and “nice” ratings in opposite-sex faces.** Preference score higher than 4.5 (depicted by a horisontal line) means that the self transform was on average preferred over the non-self transform. Error bars represent 95% CI. EMM … Estimated Marginal Means.

**FIGURE 4:**
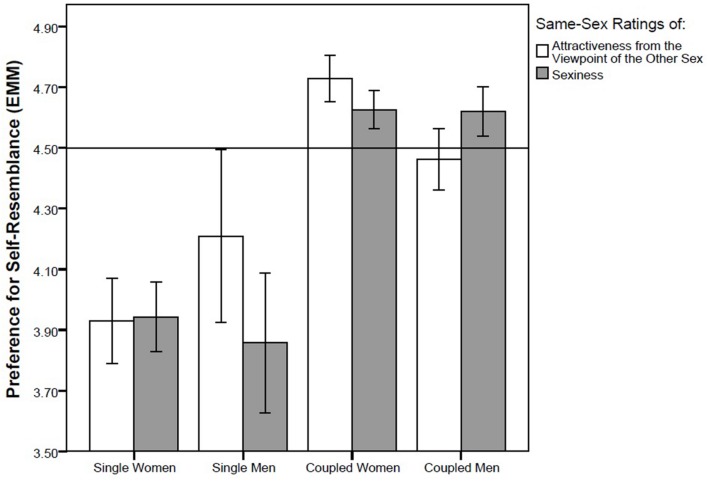
**Self-resemblance preference in ratings of attractiveness from the viewpoint of the opposite sex and sexiness in same-sex faces.** Preference score higher than 4.5 (depicted by a horisontal line) means that the self transform was on average preferred over the non-self transform. Error bars represent 95% CI. EMM … Estimated Marginal Means.

**FIGURE 5:**
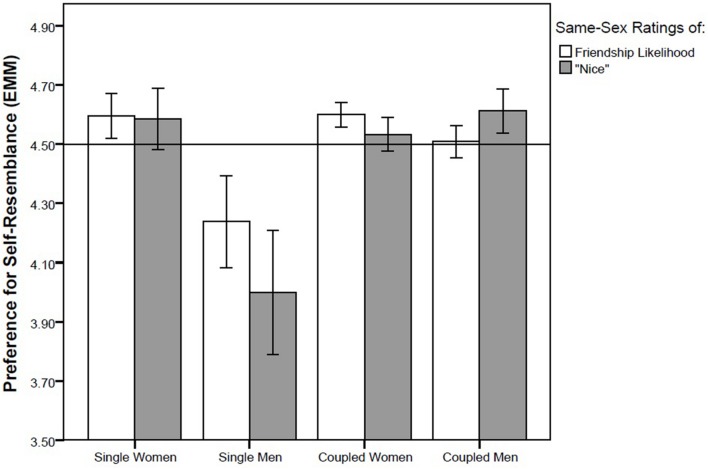
**Self-resemblance preference in ratings of long-term attractiveness and “nice” ratings in same-sex faces.** Preference score higher than 4.5 (depicted by a horisontal line) means that the self transform was on average preferred over the non-self transform. Error bars represent 95% CI. EMM … Estimated Marginal Means.

We performed four GLM analyses with different pairs of repeated measures, namely (1) self-resemblance preference in short-term attractiveness ratings of opposite-sex faces (data from Study 1) and in opposite-sex sexiness ratings (data from Study 2), (2) self-resemblance preference in long-term attractiveness rating of opposite-sex faces (data from Study 1) and “nice” rating of opposite-sex faces (data from Study 2), (3) self-resemblance preference in attractiveness ratings of same-sex faces from the viewpoint of the opposite sex (data from Study 1) and same-sex sexiness rating (data from Study 2) and (4) self-resemblance preference when rating preference of same-sex faces as possible friends (data from Study 1) and when rating how “nice” same-sex faces appear (data from Study 2). We found no effect of either repeated factor (all *F*s between 0.02 and 0.95, all *ps* between 0.33 and 0.89) which suggests that there is no difference between ratings of sexiness and short-term attractiveness, or between ratings of how nice the face appears and long-term attractiveness. In three out of these four GLM models, partnership status showed a significant effect whereas no other factor or interaction was significant. In the first GLM model, with short-term attractiveness ratings of opposite-sex faces and opposite-sex sexiness ratings entered as repeated measures and with the control factors sex of rater, partnership status, and attractiveness of rater, the effect of partnership status was significant (*F*_1,85_ = 5.35, *p* = 0.023, η^2^ = 0.059; **Figure 2**). In the second model, with the dependent repeated factor composed of the “nice” rating and long-term attractiveness rating of opposite-sex faces, partnership status was again a significant predictor (*F*_1,83_ = 4.00, *p* = 0.049, η^2^ = 0.045; **Figure 3**). A significant effect of partnership status was also found for ratings of same-sex attractiveness from the viewpoint of the other sex and same-sex sexiness rating (*F*_1,83_ = 5.54, *p* = 0.021, η^2^ = 0.062; **Figure 4**). In all three models, singles preferred relatively higher facial dissimilarity than coupled participants. There was no effect of partnership status on self-resemblance preference for combined rating of preference of same-sex face as a possible friend and “nice” rating of same-sex face (*F*_1,83_ = 0.65, *p* = 0.42, η^2^ = 0.008; **Figure 5**).

## Discussion

We found that uncoupled participants, but not those in relationships, rate dissimilar faces as more attractive and sexy than self-resembling faces. Although the effects were significant only in Study 2, where the participants rated sexiness of the stimuli, and non-significant in Study 1 where they rated attractiveness in the short-term context, the analysis performed on the combined data from both studies revealed that there was no significant effect of study, indicating that the pattern of effects were consistent (i.e., not significantly different) across Studies 1 and 2. This combined analysis, which was more robust than the separate analyses in Studies 1 and 2, as it included a repeated measure of self-resemblance preference, also confirmed that dissimilarity was significantly more preferred by uncoupled than coupled participants. Both opposite-sex and same-sex dissimilar faces were rated as more sexy by uncoupled participants in Study 2.

Although the effect of self-resemblance was stronger for the short-term attractiveness, or sexiness ratings, a similar tendency to stronger dissimilarity preference in single compared to coupled participants was found for long-term attractiveness ratings or ratings of how nice the person appears. In fact, we found no significant effect of the short- vs. long-term attractiveness context description or “sexy” vs. “nice” rating on preference for self-resemblance. We also found no significant effect of self-resemblance on trustworthiness ratings.

Our results are in line with the idea that preferences for dissimilarity might be stronger in single people who can be expected to be more actively mate-searching for a partner, possibly as an adaptation to increase heterozygosity of potential offspring connected to greater viability of heterozygous offspring or avoidance of homozygosity for deleterious genes. In contrast, this effect is diminished in coupled individuals, where other factors, such as potential kinship or friendship, may exert greater influence in facial judgments. Other studies have also found an effect of partnership status on perception of opposite-sex individuals, leading to the conclusion that coupled individuals seem to pay less attention to or derogate cues of sexual attractiveness in others compared to singles (Simpson et al., 1986; Miller, 1997; Maner et al., 2008; Lundström and Jones-Gotman, 2009; Karremans et al., 2011; Koranyi and Rothermund, 2012). As a result, coupled participants are likely to pay relatively more attention to cues of kinship or psychological similarity, of which facial self-resemblance might serve as a marker. This can also be associated with several benefits, including increased kinship support and prosocial behavior from others (Hamilton, 1964; Trivers, 1971; Bereczkei, 1998) or higher satisfaction in any kind of a dyad due to assortative pairing (Cohen, 1977; Kandel, 1978).

The possible mechanism in partnership status-related changes in facial preferences might involve testosterone levels. In general, testosterone is lower in coupled compared to single men (Booth and Dabbs, 1993; Gray et al., 2002, 2004; Burnham et al., 2003) and perhaps also women (van Anders and Goldey, 2010), being commonly interpreted as the result of higher intra- and intersex competition in singles and stronger bond maintenance in coupled people (van Anders and Watson, 2006). This is in good accord with our interpretation of the perceptual difference between single and coupled participants, where the former pay more attention to cues of attractiveness in faces of others, whereas the latter are more attentive to cues of kinship, prosociality, or social support.

The alternative hypothesis based on the assumption that coupled individuals are more likely to look for short-term extra pair partners, where cues of genetic compatibility including dissimilar appearance are considered to play a greater role, and therefore, they should prefer dissimilar faces, was not supported in our sample of participants. Future studies should test how our results can be generalized. It is still possible that coupled participants with higher extra-pair sexual interest, e.g., formally committed older adults, would show higher preferences for dissimilarity. Instead, it is likely that our sample of coupled students in their twenties often find themselves in a romantic relationship characterized by romantic love, which promotes commitment by motivating approach toward an intimate partner and countervails feelings of desire for others (Gonzaga et al., 2001).

Contrary to a previous study (DeBruine, 2005), we found no effect of the short- vs. long-term attractiveness context description. Neither did we find an effect of ratings of “sexy” vs. “nice” on preference for self-resemblance in opposite-sex faces. The effect of short vs. long-term mating context might not be strong as this task might be cognitively demanding and perhaps of low ecological validity. As argued by van Anders and Goldey (2010), people (at least in early adulthood) relatively constantly pursue a competitive or bond maintenance behavioral strategy rather than switch between them. The study of Garcia and Reiber (2008) who found that although almost 2/3 of college students have engaged in a hook-up, 1/2 of them were motivated by the intention of initiating a traditional romantic relationship, can serve as indirect evidence. In addition, differences between our findings and those of DeBruine (2005) might be related to differences in methodology used to create composite faces between the two studies. As in the majority of previous studies, DeBruine (2005) used composite images made from a relatively large number of faces, specifically, composites of 20 people of a given sex and ethnicity. Such composites are normally average, symmetrical, and attractive, and thus they form a rather specific set of target faces where self-resemblance might have a somewhat different effect from that on a sample of more widely varying individual faces. In real life, however, we perceive faces that are indeed highly variable in their morphology and texture, and where specific traits (such as, for example, cues of self-resemblance) can be more difficult to identify. In our study, we used composites drawn from only three images, which arguably produced more distinctive images and may have altered the accessibility of the task.

The new form of instructions that we used in Study 2, namely rating how “sexy” and “nice” a person appeared instead of rating attractiveness in verbally described hypothetic short-term or long-term contexts, led to a very similar pattern of results as the more typical method used in Study 1, at least regarding opposite-sex faces. However, a significant effect of partnership status on self-dissimilarity preference was found for ratings of sexiness of same-sex faces in Study 2, but not for the analogical rating in Study 1 where participants were instructed to rate attractiveness of same-sex faces from the viewpoint of the opposite sex (the effect in Study 1 was not significant). This might be considered as evidence for the suitability of the adjective ‘sexy’ for studies which employ ratings of sexual attractiveness of either opposite- or same-sex faces. Unlike the verbal descriptions, adjectives may be more easily used with same-sex photographs, and the associated rating task may more likely reflect the intuitive situation of evaluating one’s own characteristics within the mating market in which the individual exists. In a similar way to how uncoupled individuals were argued to be more sensitive to attractiveness cues in potential mates, they can be expected to be more sensitive to the cues of attractiveness of same sex individuals as potential rivals. Consequently, the effect of self-resemblance on attractiveness judgments of individuals of the same-sex might be explained by jealousy and derogation (devaluation of the attributes of a rival, Buss and Dedden, 1990), wherein self-resembling same-sex persons are rated as less attractive. This is because people tend to derogate attractiveness of potential rivals who are similar to them as they represent a stronger threat to the distinctiveness of the individual to potential partners (Broemer and Diehl, 2004).

Finally, we have not confirmed the previously found effect of self-resemblance on trustworthiness ratings (DeBruine, 2002, 2005; Hancock and DeBruine, 2003). This effect again could have been missed in our study by rating of real-looking photographs instead of composites, but there were also other differences between her study and ours. DeBruine (2002) used photographs which she transformed either regarding shape or shape and color, rather than shape only, and she used measurements of real behavior instead of reported preferences. The significant effect of self-resemblance that she reports may have been elicited as a result of this highly realistic rating situation. It is possible that when explicitly instructed to rate trustworthiness, the subjects tend more to base their judgment on common stereotypes about what trustworthy people look like and do not judge according to their individual propensity to cooperate with such a person, which would probably be more influenced by kinship cues.

## Limitations of Study

We had a relatively low number of raters in some categories which could have limited the chances of observing an effect of sex of participant. However, our main result, the effect of partnership status on self-resemblance preference, is not affected by this limitation as it remains significant both including and excluding sex of participant in the model. We also used a commonly used, but somewhat limited, morphing procedure where only the shape of the face was modified to increase or decrease resemblance with the rater. Future studies that additionally use morphing of texture, eye color and pigmentation might test relative contributions of such cues on similarity preferences. Future research should also focus more closely on the qualities of raters’ partnerships. It is possible that the level of commitment or relationship satisfaction could influence preferences for self-resemblance.

We further found that attractiveness of the raters had significant effects in our study. As Kocsor et al. (2011) show, attractiveness (other-rated) is preferred more strongly than either self-resemblance or dissimilarity. Moreover, transforming a face to resemble an attractive person apparently makes the transform more attractive. Therefore attractive raters tend to prefer self-resembling faces, primarily in the case of same-sex transforms, and the opposite is true for non-attractive raters. Moreover, we found in Study 1 that women’s, but not men’s, attractiveness was also related to partnership status, which could have lead to false positive results. However, after including raters’ attractiveness in the models, the effect of relationship status on self-resemblance preferences still remained significant.

## Conclusion

Our results provide evidence for a shift in attractiveness perceptions of opposite- and same-sex faces associated with partnership status. More specifically, dissimilar faces are rated as more attractive and sexy by uncoupled participants, but this dissimilarity preference is not apparent in coupled participants. We argue that the possible cause of such a shift is that attractiveness perception mechanisms tuned to preference for genetically suitable partners may be suppressed during romantic relationships, when preferences for more self-resembling faces are relatively important, perhaps triggered by affiliation to bearers of kinship cues.

Perceptual changes associated with the partnership status are yet to be extensively studied to learn more about all the social domains they might affect and the biological mechanisms involved. In addition to those researchers interested in cognitive and perceptual psychological processes underlying facial judgments, the topic may also be of importance within the applied psychological sciences. For example, as uncoupled young people were found to avoid kinship cues, our findings might have utility in explaining other social phenomena such as parent and adolescent disaffection.

Our modified methodology, where participants rated how “sexy” and “nice” a person appeared, generated a similar pattern of results to rating attractiveness in verbally described hypothetical short-term or long-term contexts. We then recommended that ratings of “sexy” and “nice” can be used in future research when researchers intend to compare rating of both other-sex and same-sex photographs.

## Author Contributions

Conceived and designed the experiments: JF, JH, ASL, SCR, and JL. Performed the experiments: JL, AR, ASL, and JF. Analyzed the data: JL, JF, and JH . Wrote the paper: JL, JH, and SCR. All authors listed approved the work for publication.

## Conflict of Interest Statement

The authors declare that the research was conducted in the absence of any commercial or financial relationships that could be construed as a potential conflict of interest.
